# Testing for *Mycobacterium tuberculosis* infection using the QuantiFERON-TB GOLD assay in patients with comorbid conditions in a tertiary care endemic setting

**DOI:** 10.1186/s40794-020-0102-z

**Published:** 2020-02-19

**Authors:** Kiran Iqbal Masood, Bushra Jamil, Alnoor Akber, Maheen Hassan, Muniba Islam, Zahra Hasan

**Affiliations:** 1 Department of Pathology and Laboratory Medicine, The Aga Khan University, Stadium Road, P.O.Box 3500, Karachi, Pakistan; 2 Department of Medicine, The Aga Khan University, Karachi, Pakistan

**Keywords:** Tuberculosis, QuantiFERON-TB GOLD, Co-morbids, Interferon gamma release assay, Pulmonary TB, Extra-pulmonary TB, Latent TB, Active TB, Endemic, Immunocompromised

## Abstract

**Introduction:**

There were 10 million new cases of tuberculosis (TB) in 2017. To eliminate TB, it is necessary to diagnose active TB and latent tuberculosis infection (LTBI). Diagnosis of paucibacillary disease and in extrapulmonary TB (EPTB) remains challenging; low mycobacterial load can be missed by microbiological or molecular based confirmation; EPTB, can be misdiagnosed due to absence of site specific specimens for testing. Interferon gamma release assays (IGRA) use T cell-based Interferon-gamma (IFN-γ) to identify infection with *M. tuberculosis* (MTB) but cannot discriminate between active and LTBI. We investigated how IGRA was being used in a high burden low resource setting.

**Methods:**

We conducted a retrospective review of 149 consecutive cases received for QuantiFERON-TB Gold In-Tube Assay (QFT-GIT) testing in routine clinical service.

**Results:**

Fifty-six cases were QFT-GIT positive and 93 were QFT-GIT negative. Thirty-six per cent of QFT-GIT tested cases had active TB. Of QFT-GIT positive cases, 59% patients had active TB; 10 with pulmonary and 23 with extra-pulmonary TB. The remaining 41% QFT-positive cases were LTBI. Of the QFT-GIT negative cases, 22% had active TB. Co-morbid conditions were present in 37% of QFT-GIT positive and 60% of QFT-GIT negative cases.

**Conclusions:**

Our study shows that IGRA is being used as an adjunct test for active TB in this population. It highlights the complexity of interpreting QFT-GIT results particularly for QFT-GIT negative cases when ruling out MTB infection.

## Introduction

Globally, an estimated 10 million new cases of tuberculosis (TB) were reported in 2017 [1]. Pakistan ranks 5th amongst high TB burden countries with 525,000 new cases reported [1]. It is necessary to correctly diagnose both active TB and latent tuberculosis infection (LTBI) in order to achieve the End TB target by 2035 as proposed by the World Health Organization and reduce TB incidence by 95% as compared with 2015 [2, 3].

Pulmonary disease is the predominant form of tuberculosis, although, extra pulmonary tuberculosis (EPTB) including skeletal, abdominal, genito-urinary, lymph nodes, kidney, pleural and meninges remains common [4]. Accurate diagnosis followed by appropriate management of TB is essential for controlling disease transmission. Diagnosis of pulmonary and EPTB primarily depends on a positive radiology result and microbiological confirmation through microscopy, mycobacterial culture [4] and/ or TB Xpert MTB/RIF assay (Cepheid, USA) testing. However, TB poses diagnostic dilemmas especially with regards to EPTB cases where it may not be possible to obtain an appropriate site specific specimen for laboratory confirmation or, it may be technically complex or financially unfeasible to do so.

For latent TB, the tuberculin skin test (TST) employing purified protein derivative antigen (PPD) is used most frequently [5]. Interferon gamma release assays (IGRAs) such as, QuantiFERON TB Gold in tube assay (QFT-GIT) measure specific T cell-based responses to MTB-specific antigens including Early secretory antigen target (ESAT)-6, Culture filtrate protein (CFP)-10 and TB 7.7 [6]. T SPOT-TB is an IGRA which requires purified mononuclear cells to quantify IFNgamma (IFN-γ) responses [7]. QFT- GIT uses whole blood and is more convenient to use but may be affected by the number of mononuclear cells present in the test sample [7]. QFT-GIT is more specific than TST for identification of LTBI, is unaffected by BCG vaccination [8] and can be useful as a confirmatory test to rule out false positives results [9].

A consideration in the interpretation of IGRA based results is the competence of individual immune responses. As QFT-GIT is reliant on a robust T cell response, results in young children, severely malnourished subjects or those who are HIV infected, may be compromised [10]. Immunosuppression and lymphocytopenia tend to affect results of QFT-GIT and lead to indeterminate results [11].

QFT-GIT testing has been used in differential diagnosis of active TB in multiple studies [12]. There are variable data regarding the association between QFT-GIT positive results and active TB [12]. The overall sensitivity for QFT-GIT to diagnose active tuberculosis in a meta-analysis conducted by Metcalk J et al. was found to be 69–83% [13]. Another meta-analysis conducted by Dai Y et al. [14] reported the overall sensitivity of QFT-GIT for TB diagnosis to be 85% and specificity to be 84%. Usage of QFT-GIT has been recommended as a rule out active tuberculosis in areas with low prevalence of tuberculosis [15, 16]. Due to its higher cost as compared with the TST it is not routinely recommended for screening of LTBI in high burden low middle income countries (LMIC) [17]. However, TST is recommended for screening at risk groups such as patients prior to initiation of anti-tumour necrosis factor (TNF) treatment, patients receiving dialysis, or patients preparing for organ or haematologic transplantation [5].

IGRA testing using the QFT-GIT assay was initiated at the Clinical Laboratory, The Aga Khan University Hospital (AKUH), Pakistan in 2013. We conducted this retrospective review to understand the cohort of patients in whom IGRA testing was being requested. There is currently no published data regarding the performance of IGRA testing in this population. This study reveals important insights into the context in which IGRA testing is routinely being performed and gives relevance to its utility as an adjunct test for EPTB.

## Methods

### Study subjects

This was a retrospective analysis of cases for whom QuantiFERON-TB Gold In Tube assay (QFT-GIT; Cellestis, Ltd. Germany) was performed at AKUH Clinical Laboratory, Karachi, Pakistan during the period July to December 2013. One hundred and forty-nine hospital in-patient cases for whom QFT-GIT tests were referred by physicians as per their routine laboratory work were included in the study in a consecutive sampling method. Laboratory data and hospital medical records were reviewed, demographic data was retrieved, clinical history of the patient and comorbid conditions were documented and assessed for correlation with QFT-GIT results.

### QuantiFERON-TB gold in-tube assay

One milliliter of whole blood from each study subject was added to each of the three tubes; TB antigen (ESAT-6, CFP-10 and TB 7.7), mitogen (positive control) and Nil (negative control) provided with QFT-GIT and processed as per manufacturer’s instructions. The tubes were incubated for 16 to 24 h, plasma was harvested and IFN-γ concentrations (IU/mL) in plasma was measured by an ELISA reader at 450 nm and calculated by the ‘QFT-TB-analysis Software’. A cut-off of IFN-γ ≥ 0.35 IU/ml was used. A determinate test must have mitogen minus negative control ≥0.5 IU/ml and/or TB antigens minus negative control ≥0.35 IU/ ml.

### Classification of study subjects

Cases were categorized based on a review of hospital clinical and laboratory records for each patient. Cases were grouped into those with ‘Active TB’, ‘LTBI’ and ‘Non-TB’. Cases with ‘Active TB’ were defined as cases positive for MTB culture/ acid-fast bacilli smear/ histopathology predictive of granulomatous inflammation had a strongly suggestive chest X-ray with matching clinical history and/ or responded to anti-tuberculous therapy (ATT). Patients had both ‘PTB’, and ‘EPTB’. EPTB included sites; lymph node (LN), abdominal, tuberculous meningitis (TBM), central nervous system (CNS), pelvic, pleural, peritoneal, thyroiditis, knee and spine.

LTBI were QFT-GIT positive cases with a negative diagnosis of active TB (as defined by above mentioned criteria). Non-TB cases were QFT-GIT negative with a negative diagnosis of active TB (as defined by above mentioned criteria).

### Statistical analysis

Statistical analysis was carried out using the Graphpad PRISM and Statistical Packages for Social Sciences (SPSS). Data was presented as median values. The Chi squared test was used to compare different parameters between QFT-GIT positive and negative groups.

## Results

### Characteristics of study subjects

**Fig. 1:**
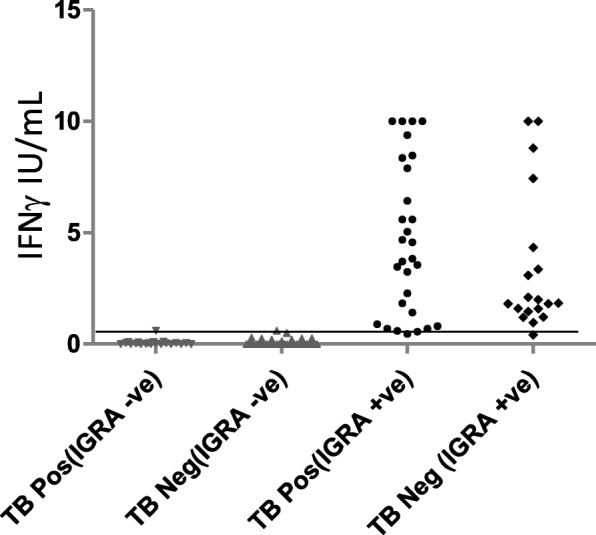
IFNγ titers in QFT-GIT negative and QFT-GIT positive TB patients. Whole blood from study subjects was incubated in QFT-GIT (NIL, TB and mitogen) tubes for 18 h. ELISA was performed to detect IFN-γ levels. Data is represented in the form of scatter plots. Horizontal line indicates cut-off at 0.35 IU/mL

Of the 149 cases we reviewed, 56 (37.5%) had a positive QFT-GIT result and 93 (62.5%) had a negative QFT-GIT result. The results of all cases are illustrated in Fig. 1. No indeterminate results were obtained. Age and gender were found to be comparable between the QFT-GIT positive and negative groups (Table 1). Nineteen cases (34%) in the QFT-GIT positive group and 37 (40%) cases in QFT-GIT negative group were observed to have lymphopenia. Further, we evaluated the cases for co-morbid conditions and identified a range of conditions such as auto-immune disease (including, Rheumatoid arthritis, Systemic Lupus Erythematosis and mixed connective tissue disorders), chronic kidney disease, malignancy, chronic liver disease, chronic obstructive pulmonary disease, achalasia, chronic heart disease, epilepsy and endocrine disorders. Of the 56 QFT-GIT positive subjects, 35 (63%) did not have any co-morbid conditions whilst 21 (27%) did so. Of the 93 QFT-GIT negative subjects, 37 (40%) did not have comorbid conditions whilst 56 (60%) had known co-morbid conditions. There were a significantly greater (*p* = 0.007) number of individuals with co-morbid conditions in the QFT-GIT negative group

**Table 1:** Baseline characteristics of study subjects

	Overall (n)	QFT positive (n)	QFT negative (n)	*P* value
Age	149	56	93	NS
Sex (female)	77	28	49	0.75
Sex (male)	72	28	44	0.75
Lymphopenia*	56	19	37	0.665
Autoimmune disease	7	0	7	< 0.001
Chronic kidney disease	7	2	5	0.613
Renal transplant	2	0	2	0.331
Malignancy	15	4	11	0.357
Chronic liver disease	2	0	2	0.331
COPD	1	0	1	0.439
Achlasia	1	1	0	0.439
Coronary heart disease	1	0	1	0.439
Diabetes	20	7	13	0.809
Other endocrine disorders	1	0	1	0.439
Epilepsy	2	1	1	0.744
Multiple co-morbids	18	6	12	0.096
No Known comorbids	72	35	37	0.007

‘*’ lymphopenia defined as lymphocytes <1500 cells/mm3; ‘QFT’, Quantiferon-Gold in Tube assay (Cellestis, Germany); Cut-off for QFT positive ≥ 0.35 IU/ml.’*’ lymphopenia is defined as lymphocytes <1500 cells/mm3; ‘#’ patients with multiple diseases including, diabetes, chronic liver disease, chronic kidney disease, endocrine disorders (panhypo-pitutiarism, hypothyroidism, thalassemia and malignancy); ‘**’ autoimmune disease includes Rheumatoid arthritis, Systemic Lupus Erythematosis and mixed connective tissue disorders. COPD, chronic obstructive pulmonary disorder. QFT negative and QFT positive groups are compared using Chi-square test. *P* value ≤ 0.05 is considered to be significant between the two groups.

More individuals in the QFT-GIT negative group had autoimmune disease (*p* < 0.001).

### Latent and active TB in QFT-GIT positive cases

Patients with active TB were identified in both QFT-GIT positive and negative cases (Table 2). Of the 56 QFT-GIT positive cases, 33 were found to have active disease and 23 were identified as LTBI. The IFNγ levels from QFT-GIT testing is illustrated in Fig. 1. The range of positive values was comparable between LTBI, PTB and EPTB groups (data not shown).

**Table 2:** Diagnostic details for patients with active TB

	N	AFBC	Microscopy	Radiology	ATT	Histo	PCR
QFT + ve							
PTB	10	4	0	10	8	2	N/A -
EPTB	23	6	0	11	14	5	1
QFT -ve							
PTB	3	1	1	2	3	0	1
EPTB	17	0	0	5	9	7	N/A

*QFT* QuantiFERON-Gold In Tube Assay, *PTB* Pulmonary TB, *EPTB* Extra-pulmonary TB, *N* Number of subjects, *AFBC* Acid fast bacilli culture by MIGIT system (Becton Dickinson, USA); Microscopy, acid fast bacillus smear using Ziehl Neelsen staining; Radiology, XRay/ CT scan/ or MRI; ATT, anti-tuberculosis treatment response; histo-pathological staining of biopsy material and or FNAC (fine needle aspirate cytology) where relevant. The numbers indicate those diagnosed as positive by each method. N/A, not available

Of the QFT-GIT positive active TB cases, these included ten PTB and twenty-three EPTB cases. The EPTB cases had TB at different sites including, lymph nodes, abdominal, TB meningitis and tuberculomas, pelvic, pleural, peritoneal, thyroid, knee and the spine (Table 3).

**Table 3:** Characteristics of study subjects

TB Status	Total (*n* = 149)	Male to female ratio M;F	Age, years (Median)	TLC (10 e^3^/L)	Lymphocyte count (%)
QFT Positive (*n* = 56)					
Active TB	(*n* = 33)				
PTB	10	3:7	44	7.9	17.25
EPTB	23	8:15	44	7.85	24.1
Latent TB	23	17:6	50	8.5	22.05
QFT Negative	(*n* = 93)				
Active TB	(*n* = 20)				
PTB	3	0:3	18	7	27.8
EPTB	17	9:8	45	9.4	17.2
Non TB	73	35:38	54	8.5	21.3

‘TLC’, total lymphocytes count; ‘QFT’, Quantiferon-Gold in Tube assay (Cellestis, Germany); ‘PTB’, pulmonary TB; ‘EPTB’, extrapulmonary TB (lymph node, abdominal, TB meningitis, tuberculoma, pelvic, pleural, thyroid, knee and spine). ‘M:F’, male: female; ‘Active TB’, subjects with a confirmed TB diagnosis based on positive radiology, microbiology, microscopy testing and/or a positive treatment response; ‘Probable ‘Latent TB’ cases were QFT-G positive without a confirmed diagnosis of TB; ‘Non-TB’, cases were QFT-G negative and without a confirmed diagnosis of TB. Cut-off for QFT positive ≥0.35 IU/ml. Lymphopenia is defined as lymphocytes < 1500 cells/mm3. Data is presented as Median values

### Active TB in QFT-GIT positive cases

Of the ninety-three QFT-GIT negative cases reviewed, 20 (21.5%) patients were found to have active TB; 3 with pulmonary and 17 with EPTB (Table 3). Follow up clinical records showed that these patients with TB were subsequently put on anti-tuberculous therapy (ATT) and all of them showed a positive response to treatment as assessed by clinical and radiological improvement at the end of treatment completion.

Seventy-three QFT-GIT negative cases who did not have signs and symptoms of active disease were categorized as being unlikely of having MTB infection, as non-TB cases.

Further, a sub-group analysis was conducted for using QFT-GIT to identify active TB in our study cohort. For this, the TB positive (IGRA negative) were taken as False Negatives (*n* = 20), TB negative (IGRA negative) as True Negatives (*n* = 93), TB Positive (IGRA positive) as True Positives (*n* = 33) and TB Negative (IGRA positive) as False Positives (*n* = 23). This sub-group analysis gave a sensitivity of 62.3% and a specificity of 76% for IGRA based identification of active TB infection in our cohort.

### Hematological characteristics and comorbid conditions

Hematological characteristics and the IFN-γ responses of QFT-GIT positive and negative groups were compared. Patients in the QFT-GIT group had a range of co-morbid conditions such as, chronic kidney disease, diabetes mellitus, malignancy, epilepsy and patients with more than one chronic illness (*n* = 21, 37.5%), Table 4. The TB antigen and mitogen results of the QFT-GIT test and lymphocyte counts for the cases sub-divided into different co-morbid conditions are illustrated in Table 4. Patients with malignancy had higher lymphocyte counts than the normal range (< 1500 cells/mm^3^) while patients with type 2 diabetes had lower lymphocyte counts than normal levels. Overall, 35 (62.5%) did not have any known co-morbid conditions.

**Table 4:** Co-morbid conditions present in QFT positive study subjects

Co-Morbid	N (%)	TB Ag (IFNγ IU/ml, Median)	Mitogen (IFNγ IU/ml, Median)	TLC (10 e3/L)	Lympho (%)
No Known comorbids	35 (62.5)	4.68	10	7.8	22.4
Diabetes	7 (12.5)	1.82	10	9.3	**15.9**^**#**^
Malignancy	4 (7)	1.975	2.96	11.3*	44.35
Achlasis	1 (1.8)	0.59	2.47	6.2	38
Chronic Kidney disease	2 (3.6)	5.91	4.795	8.8	23.3
Epilepsy	1 (1.8)	1.59	10	7	23.4
Multiple comorbids	6 (10.7)	1.51	5.78	8.2	20.5

‘TLC’, total lymphocytes count; ‘QFT’, Quantiferon-Gold in Tube assay (Cellestis, Germany). QFT results for TB antigen (TB Ag) and Mitogen are provided. Cut-off for QFT positive ≥0.35 IU/ml. ‘*’ Denotes values greater than the normal range; ‘#’ Denotes values less than the normal range. Lymphopenia defined as lymphocytes < 1500 cells/mm3. ‘#’ patients with multiple co-morbids including, diabetes, chronic liver disease, chronic kidney disease, endocrine disorders (panhypo-pitutiarism, hypothyroidism, thalassemia and malignancy)

Evaluation of QFT-GIT negative cases revealed that 39% (36 out of 93) did not have any comorbid conditions whilst 61% (57 out of 93) had a range of conditions as identified in Table 4. QFT-GIT IFN-γ responses and lymphocyte counts were compared for QFT-GIT negative cases with and without comorbid conditions for sub-groups of Active TB and Non-TB. Of the 20 cases with active TB, 8 (40%) of cases had comorbid conditions whilst 12 (60%) did not.

Sixty-seven percent (49 out of 73) of the QFT-GIT negative non-TB group had comorbid conditions. Of these, patients with diabetes had the lowest lymphocyte counts (Table 5).

**Table 5:** Co-morbid conditions present in QFT negative study subjects

Subjects with Active TB (*n* = 20)					
Co-morbids	Frequency (n, %)	TB Ag (IFNγ IU/ml, Median)	Mitogen (IFNγ IU/ml, Median)	TLC (%)	Lymphocytes (%)
None known	12 (60)	0.025	10	8.1	18.2
Diabetes	3 (15)	0.07	5.79	8.1	19.4
Other Endo	1 (5)	0.03	8.27	13.2	19
Auto immune disease^a^	1 (5)	0.01	1.85	10.7	15.9
Malignancy	1 (5)	0.04	0.77	109	1
CHD	1 (5)	0.01	10	7	32
Multiple Co-morbids^b^	1 (5)	0.1	4.08	7.7	17.7
Subjects with Non-TB (*n* = 73)					
Co-morbids	Frequency (n, %)	TB Ag (IFNγ IU/ml, Median)	Mitogen (IFNγ IU/ml, Median)	TLC (%)	Lymphocytes (%)
None known	24 (33)	0.025	10	7.4	23.6
Chronic Kidney Disease	7 (10)	0.04	8.15	7.8	20.6
Chronic Lung Disease	3 (4)	0.04	7.66	5.8	17.9
Diabetes	10 (14)	0	7.81	11	11.8
Auto Immune disease^a^	6 (8)	0.01	10	9.4	17
Malignancy	10 (14)	0.065	6.88	8.1	23.5
Epilepsy	1 (1)	0.03	10	5.8	38.4
Coronary Heart Disease	1 (1)	0	4.29	24.4	12
Multiple co-morbids	11 (15)	0	10	36	21.3

‘TLC’, Total lymphocyte count; ‘QFT’, Quantiferon TB Gold in tube assay; ‘COPD’, Chronic Obstructive Pulmonary Disorder. Cut-off for QFT positive is ≥0.35 IU/ml. Lymphopenia was determined by lymphocyte counts < 1500 cells/mm3.’^a^’ autoimmune disease includes Rheumatoid arthritis, Systemic Lupus Erythematosis and mixed connective tissue disorders. ‘^b^’ patients with multiple diseases including, diabetes, chronic liver disease, chronic kidney disease, endocrine disorders (panhypo-pitutiarism, hypothyroidism, thalassemia and malignancy)

## Discussion

The Aga Khan University Hospital Karachi, Pakistan is a tertiary care referral center for the region. The AKUH receives a large proportion of cases requiring complex clinical and surgical care. We conducted this retrospective study of samples received for routine QFT-GIT testing at the AKUH Clinical laboratory to understand how the IGRA test was performing in the cohort of cases received. This is the first such data on the utility of IGRA testing in Pakistan.

Robust identification of MTB infection is important for both latent and active disease. In endemic populations, LTBI particularly in high risk populations need to be identified and treated appropriately [17]. Key recommendations of the WHO Guidelines on management of latent tuberculosis identify groups in whom LTBI testing is strongly recommended. These include, people living with HIV, contacts of pulmonary TB cases, patients initiating anti-tumor necrosis factor therapy, patients receiving dialysis and patients preparing for transplantation [5]. Individuals with suppressed immune response are at an increased risk of developing active tuberculosis as compared to the general population [18, 19].

The QFT-GIT is a specific and convenient tool for screening MTB infection [20]. The WHO does not recommend the replacement of TST with this technology within low- and middle-income countries as it would be more costly [5]. However, QFT-GIT is more specific than TST for identification of MTB infection and therefore can reduce overestimation of infection and treatment rates [21, 22]. In 2017, QFT-GIT was replaced by the QFT Plus (Qiagen) which has two different TB antigen tubes for T cell recognition allowing further improvements in MTB specific antigen detection [23].

We found that 59% of the QFT-GIT positive cases we studied had active TB. In all, 18% of QFT-GIT cases had PTB whilst 41% had EPTB. QFT-GIT positive results in active TB are in line with previous studies which show IGRAs to have high specificity in detection of individuals with exposure to MTB [12]. For active TB case finding it is recommended that clinical signs and symptoms of TB be documented prior to testing for LTBI in addition to chest radiography. The remaining 41% of QFT-GIT positive cases were post probably LTBI where testing had been performed for various reasons such as, screening post-exposure to TB patients and unexplained febrile illness.

We investigated the QFT-GIT negative cases and found that 20 of them (22%) had Active TB. Most of these (17 of 20) had EPTB. Therefore, it appeared that here, QFT-GIT testing had been performed as an adjunct test for active TB case finding. Of the QFT-GIT negative cases, 9 were on steroid treatment, one had autoimmune disease and one had received chemotherapy. Patients with malignancy among QFT-GIT negative subjects with active TB patients had lowered lymphocyte counts than the normal range. As QFT-GIT relies on effective T cell responses, the interpretation of the test needs to take into account factors that influence host immune parameters. There is not much data available on the interpretation of QFT-GIT in patients with significant underlying illnesses. We found lymphopenia and lowered IFN-γ values to be associated with conditions such as, chronic kidney disease, renal transplant patients, diabetes, autoimmune disease, chronic liver disease, COPD, malignancy, epilepsy, chronic heart disease, multiple co-morbidities and others with no known major coexisting medical conditions. Previous studies have shown that therapeutic levels of dexamethasone reduces TB antigen-induced IFN-γ responses significantly using a QFT-GIT assay, without affecting responses of positive control [24]. There are variable results of QFT-GIT from patients who were on immunosuppressive therapies including steroids, oral immunosuppressant and biologic therapy. A negative effect of immunosuppressive therapy on the results of IGRA has also been shown in meta-analysis comprising of 71.5% of the patients who were on immunosuppressive treatment [25]. Use of corticosteroids and infliximab has shown to convert a positive QFT-GIT into negative of 30% of the study subjects tested [26]. Lowered lymphocyte counts are also found to be associated with indeterminate QFT-G results in patients with autoimmune disease [27]. Patients with lower T lymphocyte counts such immunocompromised or those with HIV infection have been shown to have false negative QFT-GIT results [28]. This is important to consider in countries where both TB and HIV are prevalent and recognition of latent as well as active TB has implications for treatment and control.

For EPTB, IGRAs have been employed as adjunct testing for investigation of TB disease and are recommended as such by ECDC and WHO guidelines [5, 17]. In our study cohort, the QFT-GIT had a specificity of 62% with a sensitivity of 76% for diagnosis of TB. In a meta-analysis of EPTB including disease at multiple sites by Fan et al., QFT-GIT was found to have a 72% specificity and 82% sensitivity for EPTB diagnosis [15]. In a meta-analysis of only tuberculosis lymphadenitis cases, QFT-GIT specificity was 81% with a sensitivity of 89% for EPTB diagnosis [29]. The QFT-GIT sensitivity and specificity we found was lower than published studies possibly because the study design was one of consecutive convenient sampling. We did not select the study subjects and standardize the inclusion criteria. Instead we included cases referred by physicians for IGRA testing. It is possible that some of the study subjects were not suitable candidates for IGRA testing and this may have reduced the sensitivity and specificity of our study.

The AKUH hospital data illustrates that of the TB patients treated, 65% of the cases had EPTB (unpublished data). Diagnosis of MTB infection in patients with EPTB in a low resource setting remains problematic due to the inaccessibility of appropriate samples for testing and also due to financial considerations. As this was a retrospective study, one limitation is that we do not know how the IGRA results were utilized in management of the patient. However, from clinical records it was apparent that some patients with a negative IGRA but who had confirmation by other methods (culture, radiological) were put on anti-tuberculous treatment and were found to be responsive to it. Given our data, it appears that QFT-GIT IGRA is being used in our setting to identify MTB infection in clinically symptomatic individuals. It is only appropriate as an adjunct test if all other clinical parameters regarding the patient, other supporting laboratory and radiological test results are also taken into consideration.

## Conclusion

As 50% of our study subjects had active TB, our study indicates that the QFT-GIT is being used as an adjunct test for diagnosis of TB in this setting. In our cohort, the specificity of QFT-GIT for diagnosis of TB was 62% with a sensitivity of 76%. The largest proportion of active TB cases had extrapulmonary TB. Given the variable impact of host immunity on IGRA test results, interpretation of QFT-GIT data for clinically symptomatic cases should be done with care, in the context of a full investigation of clinical, radiological parameters.

## Data Availability

Upon request

## References

[1] WHO. Global Tuberculosis Report, vol. 2018. Geneva: World Health Organization; 2017.

[2] WHO. Implementing the End TB strategy. Geneva: World Health Organization; 2015.

[3] WHO. The End TB Strategy. In: Organization WH. 19th ed. Geneva: World Health Organization; 2015. WHO/HTM/TB/2015.

[4] Diagnostic Standards and Classification of Tuberculosis in Adults and Children (2000). Council of the Infectious Disease Society of America, September 1999. Am J Respir Crit Care Med.

[5] WHO. Guidelines on the management of latent tuberculosis infection. Geneva: World Health Organization; 2015. 25973515

[6] Pai M, Riley LW, Colford JM (2004). Interferon-gamma assays in the immunodiagnosis of tuberculosis: a systematic review. Lancet Infect Dis.

[7] Du F, Xie L, Zhang Y, Gao F, Zhang H, Chen W (2018). Prospective Comparison of QFT-GIT and T-SPOT.TB Assays for Diagnosis of Active Tuberculosis. Sci Rep.

[8] Centers for Disease Control and Prevention (2011). Prevention NCfHVHSaT. TB Elimination: Tuberculin Skin Testing.

[9] Muñoz L, Santin M, Alcaide F, Ruíz-Serrano MJ, Gijón P, Bermúdez E (2017). QuantiFERON-TB gold in-tube as a confirmatory test for tuberculin skin test in tuberculosis contact tracing: a noninferiority clinical trial. Clin Infect Dis.

[10] Trehan I, O'Hare BA, Phiri A, Heikens GT (2012). Challenges in the management of HIV-infected malnourished children in sub-Saharan Africa. AIDS Res Treat.

[11] Kordy F, Waters V, Den-Hollander C, Read S, Lam R, Kitai I (2018). Optimizing blood collection and processing for Quantiferon-TB gold in-tube testing gives low rates of indeterminate results: clinical implications. Pediatr Infect Dis J.

[12] Trajman A, Steffen RE, Menzies D (2013). Interferon-gamma release assays versus tuberculin skin testing for the diagnosis of latent tuberculosis infection: an overview of the evidence. Pulm Med.

[13] Metcalfe JZ, Everett CK, Steingart KR, Cattamanchi A, Huang L, Hopewell PC (2011). Interferon-gamma release assays for active pulmonary tuberculosis diagnosis in adults in low- and middle-income countries: systematic review and meta-analysis. J Infect Dis.

[14] Dai Y, Feng Y, Xu R, Xu W, Lu W, Wang J (2012). Evaluation of interferon-gamma release assays for the diagnosis of tuberculosis: an updated meta-analysis. Eur J Clin Microbiol Infect Dis.

[15] Fan L, Chen Z, Hao XH, Hu ZY, Xiao HP (2012). Interferon-gamma release assays for the diagnosis of extrapulmonary tuberculosis: a systematic review and meta-analysis. FEMS Immunol Med Microbiol.

[16] Lavender TW, Barrett A, Magee J, Ong EL (2013). Interferon-gamma release assays in the diagnosis of active tuberculosis disease in a low-incident setting: a 5-year review of data. Clin Microbiol Infect.

[17] European Centre for Disease Prevention and Control (2011). Use of interferon-gamma release assays in support of TB diagnosis.

[18] Jeon CY, Murray MB (2008). Diabetes mellitus increases the risk of active tuberculosis: a systematic review of 13 observational studies. PLoS Med.

[19] Stevenson CR, Forouhi NG, Roglic G, Williams BG, Lauer JA, Dye C (2007). Diabetes and tuberculosis: the impact of the diabetes epidemic on tuberculosis incidence. BMC Public Health.

[20] Mensah GI, Sowah SA, Yeboah NY, Addo KK, Jackson-Sillah D (2017). Utility of QuantiFERON tuberculosis gold-in-tube test for detecting latent tuberculosis infection among close household contacts of confirmed tuberculosis patients in Accra. Ghana Int J Mycobacteriol.

[21] Qiu X, Tang Y, Zou R, Zeng Y, Yue Y, Li W (2019). Diagnostic accuracy of interferon-gamma-induced protein 10 for differentiating active tuberculosis from latent tuberculosis: a meta-analysis. Sci Rep.

[22] Munoz L, Santin M (2013). Interferon-gamma release assays versus tuberculin skin test for targeting people for tuberculosis preventive treatment: an evidence-based review. J Inf Secur.

[23] Theel Elitza S., Hilgart Heather, Breen-Lyles Margaret, McCoy Kevin, Flury Rhiannon, Breeher Laura E., Wilson John, Sia Irene G., Whitaker Jennifer A., Clain Jeremy, Aksamit Timothy R., Escalante Patricio (2018). Comparison of the QuantiFERON-TB Gold Plus and QuantiFERON-TB Gold In-Tube Interferon Gamma Release Assays in Patients at Risk for Tuberculosis and in Health Care Workers. Journal of Clinical Microbiology.

[24] Clifford V, Zufferey C, Germano S, Ryan N, Leslie D, Street A (2015). The impact of anti-tuberculous antibiotics and corticosteroids on cytokine production in QuantiFERON-TB gold in tube assays. Tuberculosis (Edinb).

[25] Wong SH, Gao Q, Tsoi KK, Wu WK, Tam LS, Lee N (2016). Effect of immunosuppressive therapy on interferon gamma release assay for latent tuberculosis screening in patients with autoimmune diseases: a systematic review and meta-analysis. Thorax..

[26] Edwards A, Gao Y, Allan RN, Ball D, de Graaf H, Coelho T (2017). Corticosteroids and infliximab impair the performance of interferon-gamma release assays used for diagnosis of latent tuberculosis. Thorax..

[27] Gonzalez-Moreno J, Garcia-Gasalla M, Losada-Lopez I, Cifuentes Luna C, Mir Viladrich I, Fernandez-Baca V (2018). IGRA testing in patients with immune-mediated inflammatory diseases: which factors influence the results?. Rheumatol Int.

[28] Takenami Iukary, Loureiro Camila, Machado Almério, Emodi Krisztina, Riley Lee W., Arruda Sérgio (2013). Blood Cells and Interferon-Gamma Levels Correlation in Latent Tuberculosis Infection. ISRN Pulmonology.

[29] Liu Q, Li W, Chen Y, Du X, Wang C, Liang B (2017). Performance of interferon-gamma release assay in the diagnosis of tuberculous lymphadenitis: a meta-analysis. PeerJ..

